# The viral aetiology of influenza-like illnesses in Kampala and Entebbe, Uganda, 2008

**DOI:** 10.4102/ajlm.v2i1.65

**Published:** 2013-06-24

**Authors:** Stephen Balinandi, Barnabas Bakamutumaho, John T. Kayiwa, Juliette Ongus, Joseph Oundo, Anna C. Awor, Julius J. Lutwama

**Affiliations:** 1 Field Epidemiology and Laboratory Training Program, Kenya; 2 Uganda Virus Research Institute, Uganda; 3 Department of Medical Laboratory Sciences, Jomo Kenyatta University of Agriculture and Technology, Kenya

## Abstract

**Background:**

As the threat of zoonoses and the emergence of pandemic-prone respiratory viruses increases, there is a need to establish baseline information on the incidence of endemic pathogens in countries worldwide.

**Objectives:**

To investigate the presence of viruses associated with influenza-like illnesses (ILI) in Uganda.

**Methods:**

A cross-sectional study was conducted in which nasopharyngeal swab specimens were collected from patients diagnosed with ILI in Kampala and Entebbe between 14 August 2008 – 15 December 2008. A multiplex polymerase chain reaction assay for detecting 12 respiratory viruses was used.

**Results:**

A total of 369 patients (52.3% females) was enrolled; the median age was 6 years (range 1–70). One or more respiratory viruses were detected in 172 (46.6%) cases and their prevalence were influenza A virus (19.2%), adenovirus (8.7%), human rhinovirus A (7.9%), coronavirus OC43 (4.3%), parainfluenza virus 1 (2.7%), parainfluenza virus 3 (2.7%), influenza B virus (2.2%), respiratory syncytial virus B (2.2%), human metapneumovirus (1.4%), respiratory syncytial virus A (1.1%), parainfluenza virus 2 (0.5%) and coronavirus 229E (0.5%). There were 24 (14.0%) mixed infections.

**Conclusions:**

This study identified some of the respiratory viruses associated with ILI in Uganda. The circulation of some of the viruses was previously unknown in the study population. These results are useful in order to guide future surveillance and case management strategies involving respiratory illnesses in Uganda.

## Introduction

Globally, influenza-like illnesses (ILI), also known as acute respiratory illnesses, are common causes of morbidity and mortality in both developed and developing countries.^1,2^ In temperate climates, ILI is reported throughout the year amongst hospital patients with a marked increase in cases recorded during winter periods. There is also evidence of sporadic background activity of ILI transmission throughout the year amongst communities in tropical climates, with a slight increase in cases during the rainy season.^3,4,5^ However, little is known about the aetiologic agents of ILI in some developing countries, making it a challenge to plan and implement effective patient management and disease prevention and control efforts.^6,7^

As surveillance and monitoring programmes for ILI scale up in many countries, primarily triggered by the increased threat of zoonosis and the emergence of pandemic-prone respiratory viruses, there is a need to identify and document the incidence of endemic and circulating pathogens. This information is important for differential diagnosis, outbreak investigations, trend analysis, early recognition of emerging and re-emerging viruses and implementation of specific public health interventions such as mass vaccination campaigns. In Uganda, a tropical country lying along the equator, surveillance for influenza was started in July 2007. By June 2008, influenza viruses were confirmed in only 12% of patients presenting with ILI at health facilities, implying that the aetiologies in the remaining 88% were unknown.^8^ There are a few studies in Uganda that have identified influenza viruses, respiratory syncytial viruses, parainfluenza viruses, coxsackieviruses and echoviruses as being causative agents of ILI – but these studies were conducted during the 1970s.^9,10,11^ More recent results from other countries including Senegal, Cote d’Ivoire, Kenya and Madagascar have shown that these viruses are in circulation together with newly-discovered viruses such as coronaviruses, bocaviruses and polyomaviruses.^12,13,14,15,16^ In the current study, we identify the respiratory viruses that are associated with ILI patients seeking healthcare in Kampala city and Entebbe town, both located in the central region of Uganda.

## Research methods and design

### Study locations and target population

This study was carried out under the national protocol for the routine surveillance of human influenza in Uganda. Patients of all ages presenting with ILI at the sentinel surveillance sites for influenza at Kiswa Health Centre in the Ugandan capital Kampala and Entebbe Hospital in Entebbe town were enrolled into this cross-sectional study between August and December 2008. These two study sites are separated from each other by approximately 35 km.

Using a World Health Organization (WHO)-modified criterion as used by others,^17,18,19^ an ILI case was defined as any individual presenting with fever (≥ 38 °C) and any two of the following clinical signs: cough, sore throat, myalgia and headache. A maximum of five eligible cases was enrolled on each day at each of the study sites. Patient demographic characteristics and clinical history were recorded on a standardised clinical form. The presentation of other related symptoms, such as shortness of breath, conjunctivitis, diarrhoea and vomiting, was also recorded. This information was obtained from the caregiver if the patient was unable to speak or was a child.

### Laboratory testing

All the laboratory testing was carried out at the National Influenza Centre (NIC) located at Uganda Virus Research Institute, Entebbe, Uganda. This laboratory is the national reference centre for human influenza surveillance in Uganda and participates routinely in the WHO External Quality Assessment Panel Programme for influenza viruses. A nasopharyngeal swab (Pur-Wraps^®^, Puritan Medical Products Company LLC, Maine, USA) was collected from each study participant. Specimens collected at health centres were placed immediately in 500 µl of viral transport media (Dulbecco’s Modified Eagle’s Medium^®^, Highveld Biological Ltd, Lyndhurst, South Africa; supplemented with 0.5% serum albumin, 100 U/ml of Penicillin and 100 U/ml of Streptomycin), stored in liquid nitrogen and transported to the NIC for further analysis.

A commercially-available multiplex PCR kit (Seeplex^®^ RV Detection; Seegene Inc, Rockville, MD, USA) for the detection of adenovirus, influenza A and B viruses, respiratory syncytial viruses A and B, human metapneumovirus, parainfluenza viruses 1, 2 and 3, rhinovirus A, and coronaviruses 229E and OC43 was used. This kit has been used elsewhere to identify respiratory viruses in similar specimens.^20,21^ The assay was performed according to the manufacturer’s instructions. Briefly, total nucleic acids (both ribonucleic acid [RNA] and deoxyribonucleic acid [DNA]) were extracted from the collected specimens (Viral Gene-Spin^TM^, iNtRON Biotechnology, Gyeonggi-do, Korea) and target sequences present in the extracts were then reverse transcribed (for the RNA form) and amplified in two separate assays of multiplex polymerase chain reaction (PCR; mPCR) using virus-specific kit primers. One assay contained primers for adenovirus (amplicon size 534 bp), human metapneumovirus (469 bp), coronavirus 229E (375 bp), parainfluenza virus 1 (324 bp), parainfluenza virus 2 (264 bp), and parainfluenza virus 3 (219 bp), whilst the other mPCR assay had primer sets for influenza A virus (513 bp), influenza B virus (455 bp), respiratory syncytial virus B (391 bp), rhinovirus A (337 bp), respiratory syncytial virus A (273 bp) and coronavirus OC43 (231 bp). The amplified products were observed using agarose gel electrophoresis. Specimens that had matching bands corresponding to the expected amplicon sizes for each type of virus relative to the molecular weight marker (Seeplex^®^) were scored as positive for that virus. Kit positive and negative controls were also included in every assay run. An internal positive control (Seeplex^®^) was also included to evaluate the amplification efficiency of each tested specimen.

## Ethical considerations

As these data were collected from patients as part of routine healthcare delivery and were anonymous, the Uganda Ministry of Health guidelines did not require ethical review. Informed (verbal) consent was obtained from each study participant or caregiver.

## Trustworthiness

This study was conducted following the national guidelines for Influenza surveillance as established by the Ugandan Ministry of Health. Thus, all the study participants were identified and recruited by trained healthcare workers using a modified WHO case definition for ILI. In addition, all laboratory procedures were performed in a WHO collaborating reference laboratory following the kit manufacturer’s instructions. Both the specimen collection and laboratory testing methods have been used elsewhere in similar studies.^17,18,19,20,21^

## Results

### Demographic and clinical characteristics of study participants

Between August and December 2008, a total of 369 study participants presenting with ILI at the two study centres were recruited. Of these, 286 (77.5%) participants were from Entebbe Hospital and 83 (22.5%) participants were from Kiswa Health Centre (Table 1). Both genders were represented in almost equal proportions (52.3% females and 47.7% males) and the median age was 6 years (range: 1–70). Over half of the study participants (61.5%) were aged 10 years or less, and had low or no form of education (74.5%).

**TABLE 1:** Demographic characteristics of patients with influenza-like illness at Kiswa Health Center and Entebbe Hospital, Uganda, between August 2008 and December 2008.

Characteristic	Variable	Entebbe Hospital	Kiswa Health Center	Overall (%)
		*n* = 286	*n* = 83	*n* = 369
Gender	Male	133	43	176 (47.7)
	Female	153	40	193 (52.3)
Median Age (Years)	-	7	3	6
Age-groups (Years)	<1	37	9	46 (12.5)
	1–5	86	43	129 (35.0)
	6–10	37	15	52 (14.0)
	11–20	40	8	48 (13.0)
	21–30	54	5	59 (16.0)
	31+	32	3	35 (9.5)
Maximum level of education completed	None/Pre-primary	205	70	275 (74.5)
	Primary	57	7	64 (17.3)
	Secondary	21	6	27 (7.3)
	Tertiary	3	0	3 (0.8)
Occupation	Child/Student	186	71	257 (69.6)
	Unemployed	75	4	79 (21.4)
	Self-employed	20	7	27 (7.3)
	Private/Government-employed	5	1	6 (1.6)

*Source*: Authors’ own construction

All patients were seen at the outpatient departments of the two study sites and none required hospital admission at the time of enrolment. Apart from fever, the most common clinical symptoms were cough (98.4%), shortness of breath (43.1%) and headache (29.0%). Only 3.8% (*n* = 14) of the study participants reported the presence of a chronic condition or illness such as active tuberculosis, chronic cough and chest pain. Almost all of the participants (94.5%) reported to the clinic within three days (range: 1–31) of the onset of symptoms.

### Frequencies of detected viral aetiologies

Out of 369 swab specimens collected and analysed, 172 (46.6%) were positive for one or more respiratory agents. The most frequently-detected respiratory virus amongst all study participants was influenza A (*n* = 71, 19.2%). Others were adenovirus (*n* = 32, 8.7%), rhinovirus A (*n* = 29, 7.9%) and coronavirus OC43 (*n* = 16, 4.3%). The least-detected respiratory viruses were parainfluenza virus 2 (*n* = 2, 0.5%) and coronavirus 229E (*n* = 2, 0.5%) (Table 2). Approximately 63.0*%* of all viral detections, including all the parainfluenza viruses (1, 2 and 3) and 78.1% of adenovirus and 75.0% of coronavirus OC43, were detected from study participants who were aged 10 years or less. Only Rhinovirus A and all influenza viruses were detected across all ages.

**TABLE 2:** Frequency and prevalence of respiratory viruses amongst patients presenting with influenza-like illness at Kiswa Health Center and Entebbe Hospital, Uganda, between August 2008 and December 2008; *n* = 369.

Detected respiratory virus	Frequency	Prevalence (95% CI)
Influenza A virus	71	19.2 (15.7 – 24.0)
Adenovirus	32	8.7 (6.1 – 12.1)
Rhinovirus A	29	7.9 (5.4 – 11.2)
Coronavirus OC43	16	4.3 (2.6 – 7.1)
Parainfluenza virus 1	10	2.7 (1.4 – 5.1)
Parainfluenza virus 3	10	2.7 (1.4 – 5.1)
Influenza B virus	8	2.2 (1.0 – 4.4)
Respiratory syncytial virus B	8	2.2 (1.0 – 4.4)
Human metapneumovirus	5	1.4 (0.5 – 3.3)
Respiratory syncytial virus A	4	1.1 (0.3 – 2.9)
Parainfluenza virus 2	2	0.5 (0.1 – 2.2)
Coronavirus 229E	2	0.5 (0.1 – 2.2)

*Source*: Authors’ own construction

### Mixed viral infections

Of the 172 that tested positive for respiratory agents, there were 24 (14.0%) cases with mixed infections of two or three viruses. No patient was infected with more than three viruses. Twenty-one cases were infected with two viruses, with the most frequent mixture being adenovirus and influenza A virus (*n* = 5). Other frequent viral mixtures were adenovirus and rhinovirus A (*n* =3) and influenza A virus and coronavirus OC43 (*n* =3) (Table 3). Adenovirus was present in 62.5% (*n* = 15) of mixed infections which was almost a half (46.9%) of all detections for this virus. Respiratory syncytial virus B was the only aetiology that was not detected in a mixed infection.

**TABLE 3:** Mixed infections amongst patients presenting with influenza-like illness attending Kiswa Health Center and Entebbe Hospital, Uganda, between August 2008 and December 2008; *n* = 24.

Mixed viral aetiologies	Frequency (*n*)
Adenovirus and influenza A virus	5
Adenovirus and rhinovirus A	3
Influenza A virus and coronavirus OC43	3
Adenovirus and parainfluenza virus 1	2
Influenza A virus and rhinovirus A	2
Human metapneumovirus and rhinovirus A	1
Adenovirus and human metapneumovirus	1
Parainfluenza virus 1 and parainfluenza virus 3	1
Respiratory syncytial virus A and parainfluenza virus 2	1
Adenovirus and influenza virus B	1
Influenza A virus and coronavirus 229E	1
Triple aetiologies, Parainfluenza virus 1, adenovirus and coronavirus OC43	1
Triple aetiologies, Parainfluenza virus 3, adenovirus and coronavirus OC43	1
Triple aetiologies, Parainfluenza virus 1, adenovirus and rhinovirus A	1

*Source*: Authors’ own construction

## Discussion

This study identified viral aetiologies in 46.6% of all ILI cases at two health facilities in Kampala and Entebbe, Uganda, a prevalence level similar to that reported in other studies.^22,23,24^ The identified aetiologies include influenza A and B virus, adenovirus, rhinovirus A, coronavirus OC43 and 229E, parainfluenza virus 1, 2 and 3, respiratory syncytial virus A and B and human metapneumovirus. A number of these aetiologies have only recently been identified and their circulation in Uganda was unknown previously. These include human metapneumovirus, whose discovery as a causative agent for ILI has been recognised only in the last decade.^25^

Most of the detected viruses, including parainfluenza virus 1, 2 and 3, influenza B virus, adenovirus, human metapneumovirus and coronaviruses OC43 and 229E, were circulating at prevalence levels that were, in general, similar to those found elsewhere.^12,14,26,27^ Influenza A virus was detected in 19.2% of ILI cases, which was higher than the 12% prevalence level that was known to exist from previous observations in the same population.^8^ The higher prevalence observed for this virus could be attributed to the timing of this study which was conducted when rainfall is highest in Uganda. It is probable that an outbreak associated with this virus was ongoing during the study period as observed previously.^4,5,6^ Conversely, rhinovirus A was detected at 7.9% which is lower than the 10 % – 25% prevalence levels found in other ILI surveillance studies within sub-Saharan tropics.^13,14,16,28^ In the same studies, the prevalence of respiratory syncytial viruses A and B ranged between 5% – 21% which is higher than our 3.3% total prevalence for the same viruses. The low prevalence levels observed for these viruses could also be associated with their seasonality in the study population – a variable that could not be established with our current cross-sectional data. Also, our ILI case definition was focused more on influenza surveillance and could have been restrictive with regard to the signs and symptoms of other ILI aetiologies.^29,30^

Mixed infections amongst all cases that tested positive for respiratory agents accounted for 14.0% of the findings, with the majority being double infections. The prevalence of mixed infections ranging from 4.5% – 70% are reported from other studies, depending on the geographical location of the study area, the diagnostic method used or the general degree of illness in the study population.^24,31,32,33^ The high prevalence of mixed influenza A virus and adenovirus infections during a low circulation cycle of respiratory syncytial virus infection has previously been suggested.^34^ In our study, the number of mixed infections (*n* = 24) was not adequate to allow statistical analysis; a more comprehensive study is necessary in order to determine the associations and interactions between these viruses as well as the related clinical outcomes.

## Limitations of the study

This study had a number of limitations. Firstly, the number of viruses detectable by the multiplex PCR kit was limited to 12; it is possible that other respiratory viruses were circulating in the study population but were not identified. Secondly, only nasopharyngeal swabs were collected, possibly missing viruses in the lower respiratory tract. Thirdly, the study period was limited to four months and epidemiological aspects associated with these viruses such as seasonality could not be established.

## Conclusion

We have shown the viral aetiologies of some of the ILI in the study population that were not caused by influenza viruses. This information is vital for use in the future to guide policymaking on respiratory disease surveillance and case management in Uganda.
